# Virus Pathogen Database and Analysis Resource (ViPR): A Comprehensive Bioinformatics Database and Analysis Resource for the Coronavirus Research Community

**DOI:** 10.3390/v4113209

**Published:** 2012-11-19

**Authors:** Brett E. Pickett, Douglas S. Greer, Yun Zhang, Lucy Stewart, Liwei Zhou, Guangyu Sun, Zhiping Gu, Sanjeev Kumar, Sam Zaremba, Christopher N. Larsen, Wei Jen, Edward B. Klem, Richard H. Scheuermann

**Affiliations:** 1 J. Craig Venter Institute, 10355 Science Center Dr., San Diego, CA 92121; Email: bpickett@jcvi.org (B.E.P.); dgreer@jcvi.org (D.S.G.); yun.zhang@jcvi.org (Y.Z.); lstewart@jcvi.org (L.S.); 2 Northrop Grumman Health Solutions, 2101 Gaither Rd., Rockville, MD 20850; Email: liwei.zhou@ngc.com (L.Z.); zhiping.gu@ngc.com (Z.G.); sanjeev.kumar@ngc.com (S.K.); sam.zaremba@ngc.com (S.Z.); wei.jen@ngc.com (W.J.); ed.klem@ngc.com (E.B.K.); 3 Vecna Technologies, 6404 Ivy Lane Suite 500, Greenbelt, MD 20770; Email: gsun@vecna.com (G.S.); clarsen@vecna.com (C.L.)

**Keywords:** virus, database, bioinformatics, Coronavirus, SARS, SARS-CoV, Coronaviridae, comparative genomics

## Abstract

Several viruses within the *Coronaviridae *family have been categorized as either emerging or re-emerging human pathogens, with Severe Acute Respiratory Syndrome Coronavirus (SARS-CoV) being the most well known. The NIAID-sponsored Virus Pathogen Database and Analysis Resource (ViPR, www.viprbrc.org) supports bioinformatics workflows for a broad range of human virus pathogens and other related viruses, including the entire *Coronaviridae *family. ViPR provides access to sequence records, gene and protein annotations, immune epitopes, 3D structures, host factor data, and other data types through an intuitive web-based search interface. Records returned from these queries can then be subjected to web-based analyses including: multiple sequence alignment, phylogenetic inference, sequence variation determination, BLAST comparison, and metadata-driven comparative genomics statistical analysis. Additional tools exist to display multiple sequence alignments, view phylogenetic trees, visualize 3D protein structures, transfer existing reference genome annotations to new genomes, and store or share results from any search or analysis within personal private ‘Workbench’ spaces for future access. All of the data and integrated analysis and visualization tools in ViPR are made available without charge as a service to the *Coronaviridae* research community to facilitate the research and development of diagnostics, prophylactics, vaccines and therapeutics against these human pathogens.

## 1. Introduction

The human population is constantly being barraged by newly emerging and re-emerging viral pathogens, as evidenced by sporadic outbreaks of a variety of different viruses that have occurred in recent years [1,2,3,4]. Between late 2002 and 2004, the Severe Acute Respiratory Syndrome Coronavirus (SARS-CoV), a member of the *Coronaviridae *family in the *Nidovirales *order, emerged as a new human pathogen, causing a worldwide epidemic with more than 8000 infections and 700 known deaths [5,6]. While the primary host reservoir responsible for harboring the immediate ancestor of SARS-CoV has yet to be identified, evidence of direct transmission of the virus between civets in close proximity to humans has been observed and reported in at least two distinct events [7,8,9]. 

The World Health Organization (WHO) became involved in responding to this outbreak by using modern communications technologies to establish a worldwide Collaborative Multi-Centre Research Project on Severe Acute Respiratory Syndrome (SARS) Diagnosis tasked with identifying the etiological agent of the outbreak. Multiple labs worked concurrently to determine the sequence of this new pathogen [10], investigate the structure of virions by electron microscopy [11], and generate other data that contributed to the correct identification of the causative agent [12]. Meanwhile, public health specialists implemented control measures and mitigation procedures to prevent the spread of the disease [13].

In order to support research focused on these newly emerging pathogens, the Division of Microbiology and Infectious Diseases (DMID) of the National Institute of Allergy and Infectious Diseases (NIAID), at the US National Institutes of Health (NIH), is supporting a variety of resources for researchers (http://www.niaid.nih.gov/labsandresources/resources/dmid/Pages/default.aspx) including the Genomic Sequencing Centers for Infectious Diseases, Structural Genomics Centers for Infectious Diseases, Systems Biology for Infectious Diseases Research [14], and BEI Resources Repository programs to increase public availability of genome sequence data, 3D protein structures, systems biology models, isolated organisms and experiment reagents. The Bioinformatics Resource Centers (BRC) for Infectious Diseases [15,16,17] were created to serve as database resources for the integration of research and surveillance data being generated by these DMID-sponsored resources and by other primary investigators working on infectious diseases caused by different groups of human pathogens and their insect vectors. The objective of the BRC program is to provide a one-stop-shop for data and analytical tools to support data mining and analysis workflows for both basic and applied research. The Virus Pathogen Database and Analysis Resource (ViPR) BRC (www.viprbrc.org) supports virology researchers studying select agents and other significant public health pathogens belonging to 14 virus families, including *Coronaviridae* [16]. Cross-referencing data and integrating computational tools into the online ViPR resource allows complex analyses to be easily performed by researchers regardless of their bioinformatics training or expertise.

## 2. Results and Discussion

### 2.1. ViPR Overview

**Figure 1:**
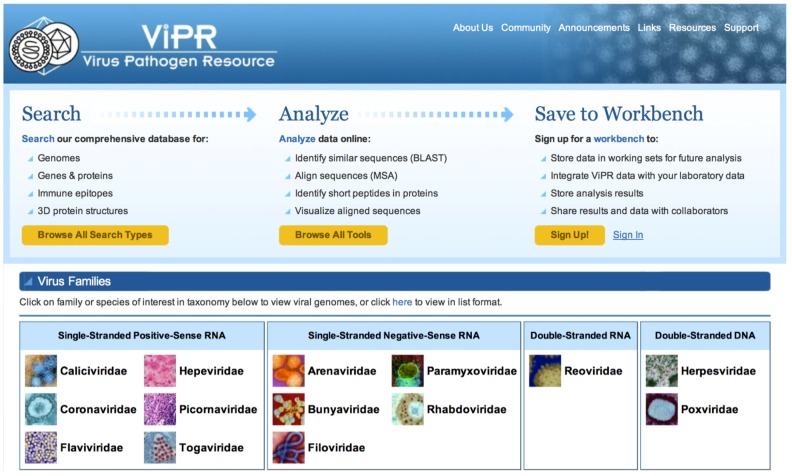
Virus Pathogen Database and Analysis Resource (ViPR) Homepage. ViPR serves as a gateway to search for information compiled from multiple sources, perform bioinformatics analyses, visualize data, and save results within the Workbench feature for the 14 virus families supported in the system, including *Coronaviridae*.

The layout of the ViPR home page, at www.viprbrc.org, highlights the three major functions of the resource (Figure 1). First, ViPR captures different types of data from both external and internal sources and makes them accessible through custom search pages. ViPR stores these data for virus families categorized as containing either human priority pathogens or possible public health threats including 6 families of single-stranded positive-sense RNA viruses—*Caliciviridae, Coronaviridae, Flaviviridae, Hepeviridae, Picornaviridae, Togaviridae*, 5 families of single-stranded negative-sense RNA viruses—*Arenaviridae, Bunyaviridae, Filoviridae, Paramyxoviridae, Rhabdoviridae*, 1 family of double-stranded RNA viruses—*Reoviridae*, and 2 families of double-stranded DNA viruses—*Herpesviridae, Poxviridae*. Although ViPR is focused on supporting human infectious disease research, related viruses within these families isolated from other host species are also accessible to allow comparative genomics research. Second, ViPR has assembled a suite of data analysis and visualization tools so that users can perform custom correlative analyses. Third, datasets and analysis results can be saved in private workspaces in the ViPR Workbench for subsequent retrieval and sharing. 

### 2.2. Data Contained in ViPR

ViPR strives to integrate data from three types of sources: (i) data transferred to ViPR from public archives, (ii) novel data derived by ViPR using a variety of computational algorithms and bioinformatics methods, and (iii) data submitted directly to ViPR by independent investigators. These data are stored in a relational database to facilitate rapid retrieval through user-specified queries.

#### 2.2.1. Data from Public Archives

ViPR aims to provide a single resource to access multiple types of data from various public resources for the virus research community. ViPR stores sequence records, manually-curated immune epitopes, 3D protein structures, and other types of public records, together with the relevant metadata (i.e. structured information associated with a given record) from GenBank [18], UniProt [19], Protein Databank (PDB, http://www.rcsb.org/pdb) [20], Immune Epitope Database (IEDB, www.IEDB.org) [21], PubMed and Gene Ontology Consortium (GO, www.geneontology.org) [22]. All of these data are regularly updated and are easily retrievable by selecting search criteria within the intuitive web-based user query interfaces implemented throughout ViPR. For *Coronaviridae*, there are currently sequence and related data from 8635 different virus strains in ViPR as of September 2012 (Table 1).

#### 2.2.2. Novel Derived and Predicted Data

Following import into ViPR, these public data are further processed to produce novel derived data through the use of various automated bioinformatics and comparative genomics algorithms implemented by ViPR and run behind the scenes, as well as information from manual curation. Such pre-calculated data includes: molecular weight, isoelectric point, and Pfam and other domains/motifs determined using InterProScan [23] for all proteins; predicted CD8+ T-cell epitopes using the NetCTL algorithm [24]; nearest BLASTp hits; predicted ortholog groups using OrthoMCL [25]; and all linked PubMed references (Table 1).

ViPR utilizes the National Center for Biotechnology and Information (NCBI) RefSeq strains [26] to extend the manually-curated RefSeq annotations to the rest of the strains belonging to the same taxon. Strain records that lack protease cleavage site information for nonstructural proteins are analyzed using custom prediction pipelines that use multiple sequence alignment to map homologous sequence regions and cleavage sites from closely-related RefSeq sequences. Sequence-based methods are also used to construct virus ortholog groups and the associated annotations, which are provided throughout the resource to easily identify proteins having similar function within the virus family even when the gene/protein names or symbols do not match. In an effort to add information that is not included in the GenBank records, ViPR has also manually-curated the scientific literature to glean information regarding the country, year and host of isolation for many of the clinically-relevant SARS-CoV strains.

Recently, we have implemented a Sequence Feature Variant Type component within ViPR that captures the location of characterized regions found in viral proteins. While this functionality is based on previous work in human HLA and influenza virus proteins [27,28], it has been extended to the various taxa within ViPR. Sequence Feature (SF) definitions are obtained from the scientific literature, GenBank, UniProt and IEDB records, and are categorized as structural (e.g. alpha-helices), functional (e.g. active sites), immune epitopes or sequence alteration SF types. SF definitions are currently available in ViPR for Dengue (serotypes 1-4), Hepatitis C (subtype 1a) and Pox (Vaccinia) viruses; however, functionality exists that allows researchers to add new definitions for any other virus species in ViPR through community annotation efforts. Such crowd-sourcing efforts are essential to provide the custom sequence feature definitions for each taxon, which can then be useful in performing data mining, comparative genomics and/or evolutionary analyses by the community as a whole. In order to maintain highly accurate definitions within ViPR, all community-proposed SFs are inspected and validated by domain experts prior to becoming publicly accessible through ViPR.

For each SF, all ViPR amino acid sequence records for viruses in the same taxon are searched in order to identify all unique sequence variations observed. All strains containing the same sequence variation pattern are then assigned to the same Variant Type (VT) category. Each defined SF has a dedicated Sequence Feature Details page in ViPR that displays information about the protein and strain from which the region was characterized and reported, the reference citations, all observed VTs, a list of all strains bearing each of the VTs, hyperlinks to any homologous 3D protein structures, and a search interface for finding a particular VT based on the user-input sequence. By performing comparative genomics analyses on unique sets of sequence variations in the short, well-characterized regions found in the SFVT component of ViPR, researchers can easily identify candidate positions that correlate with a given phenotype at a finer level of granularity and with less noise than is possible using whole-protein sequence analysis approaches.

#### 2.2.3. Data from Direct Submission

Outside institutions and programs, including the NIAID-funded Genome Sequencing Centers (GSC) for Infectious Diseases, submit *Coronaviridae* sequence metadata that may not be available in the corresponding GenBank sequence record directly to ViPR. Sequence metadata can be used in various ViPR search interfaces as query criteria to identify all strain records and/or genome sequence data that match the specified constraints, and in selected comparative genomics analysis tools.

Data from experiments interrogating host genes and proteins that respond to viral infection are currently being generated and submitted by laboratories associated with the NIAID-supported Systems Biology for Infectious Diseases Research program and the BRC Driving Biological Projects program, and have recently been included within the new host factor component in ViPR. The intent of these studies is to identify host factors that positively- and/or negatively influence virus replication across a range of experimental variables using high-throughput “-omics” methods including gene expression microarrays, proteomics and RNA interference technologies. For each set of experimental variables, the lists of host factors, called “biosets”, that are identified as being significantly different when compared to controls are submitted to ViPR. The aim of this component is to provide easy access to the processed data, establish an automated approach for users to perform a rapid comparison of their own gene(s) of interest against those that have previously been identified through virus-host response experiments, and to offer the analytical tools necessary to interpret such data. Currently, ViPR contains data from multiple host factor experiments involving SARS-CoV and influenza A virus infections in cell culture and in experimental animals, and provides a Boolean search function to identify shared, unique or combined lists of factors found to be significant between such experiments. Additional datasets from experiments using other viruses will be imported in the future.

#### 2.2.4. Search Capabilities in ViPR

Access to data in ViPR, together with the integrated analysis and visualization tools begins by clicking on the *Coronaviridae *family name on the ViPR home page. The system was designed to separate data by family to ideally manage the genome structure, data requirements and other specific nuances that are unique to each virus family. Constructing a custom query for any data type, including sequence records, is both fast and easy using the custom interfaces designed to reflect the contents in the ViPR system. Whole and partial genome sequence records can be searched with user-specified criteria including genera, species, virus host, and geographical and/or temporal point of isolation. Currently, the *Coronaviridae* component in ViPR contains sequence data for viruses annotated as being isolated from 68 different host types across 59 countries between 1941 and 2011. Keyword searches can be used to retrieve database entries based on any available metadata. More advanced pattern-matching search capabilities also exist to retrieve sequence records that contain specified sequence strings. Search results are reported in tabular format on a Genome Search Results page, which provides hyperlinks to Strain Details pages for each respective strain.

**Table 1:** *Coronaviridae* data currently provided within the ViPR database.

Data Source	Data Type	Number of Records*
Imported from Public Archives	NCBI Virus Species	384
	Virus Strains	8,635
	Genome Sequences	10,729
	Complete Genomes	615
	Unique UniProt Proteins	11,171
	Genes/Proteins	20,531
	Unique Protein Annotations	2,040
	Unique Gene Ontology Identifiers	134
	3D Protein Structures	197
	Immune Epitopes	2,253
ViPR-Generated	Pfam Domains	8,232
	Other Domains/Motifs	5,297
	PubMed References	3,969
	Predicted Ortholog Groups	50
	Predicted Mature Peptides	5,413
	Predicted CD8 Epitopes	5,116
	Nearest BLASTp Hits	18,977
Direct Submission	Host Factor Experiments	8
	Biosets	77

* Number of records in ViPR as of September 2012.

**Figure 2:**
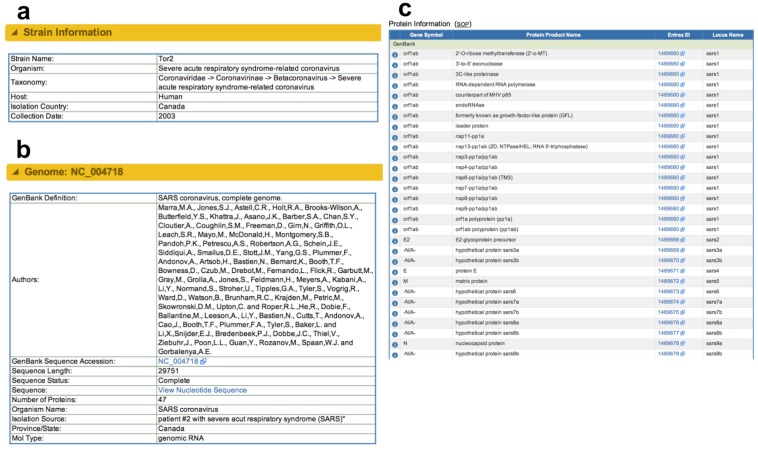
Strain and Genome Information in ViPR. The relevant strain- and genome-level annotations for the SARS-CoV Tor2 strain are parsed from the corresponding GenBank file and organized into intuitive categories for display on the ViPR Strain Details page. (a) Strain-level information includes strain name, virus taxonomy and host, country, and date of specimen isolation. (b) Information at the genome level comprises publication information, GenBank accession number, sequence length, nucleotide sequence of the genome, number of annotated proteins, and molecule type. (c) Annotated gene symbols, protein product names, Entrez ID and locus name for each gene in the genome are similarly parsed from the GenBank record and displayed in a tabular format.

**Figure 3:**
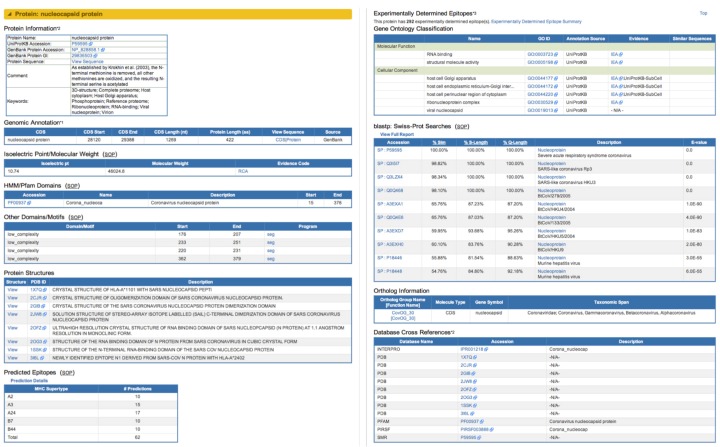
Gene and Protein Information in ViPR. The Gene/Protein Details page combines annotations at the gene and protein levels and presents them in a single comprehensive page in ViPR according to the source of the information. The information associated with the nucleocapsid protein from the SARS-CoV Tor2 strain is shown, including information such as UniProtKB and GenBank protein accession numbers, the corresponding protein sequence, genomic location, isoelectric point, molecular weight, Pfam (and other) domains, relevant 3D protein structures, predicted epitopes, experimentally-determined epitopes, Gene Ontology classification, results from BLASTp searches, and ortholog information, which are derived from UniProt, GenBank, InterProScan algorithm¬¬, PDB, NetCTL algorithm, Immune Epitope Database, Gene Ontology Consortium, BLAST, and the OrthoMCL algorithm, respectively.

The Strain Details page (Figure 2) is specific to each virus strain and contains various annotations, including strain name and taxonomy, as well as a description of the host and geo-temporal point of specimen isolation. Genome-level information such as GenBank record name and accession number, sequence length and number of proteins encoded by the genome are also displayed on the Strain Details page together with Genome Image Map graphics and Protein Information tables. Choosing a specific protein from either the graphic or the table will load a Gene/Protein Details page (Figure 3) that displays additional information obtained from public archives, including experimentally-determined immune epitopes and GO annotations, and data derived from ViPR analysis procedures, including predicted molecular weight, isoelectric point, domains/motifs, predicted immune epitopes, BLASTp results and ortholog information.

### 2.3. Analytical and Visualization Capabilities in ViPR

Once the desired sequence records are obtained from the search results page, an integrated collection of tools is provided to perform various comparative genomics analyses and visualization tasks. The suite of tools currently available in ViPR includes: multiple sequence alignment (MSA) calculation using either MUSCLE (with UCLUST) or MAUVE [29,30,31], MSA visualization and modification with JalView [32], MSA format conversion with ReadSeq [33], phylogenetic tree reconstruction using the FastME, PhyML or RAxML algorithms [34,35,36], evolutionary model selection using either modelCompare or ProtTest [37], phylogenetic tree visualization and manipulation with Archaeopteryx [38], 3D protein structure visualization and exploration using Jmol [39], metadata-driven comparative genomics statistical analysis using meta-CATS, nucleotide and amino acid sequence search against custom databases using BLAST [40], sequence variation calculation using the Weblogo algorithm [41], short peptide search with exact, fuzzy or pattern match, annotation mapping for new sequences using the Genome Annotation Transfer Utility [42], and custom PCR primer design using the Primer3 algorithm [43] (Table 2). The use of a subset of these tools will be described in more detail as part of an exploratory scientific use case described below.

**Table 2:** Analytical and visualization tools integrated into ViPR.

Tool Name	Function / Purpose
MUSCLE	Calculate a multiple sequence alignment (MSA) using either nucleotide or amino acid sequences
ReadSeq	Convert between various MSA formats
JalView	Visualize and modify nucleotide or amino acid MSA
FastME, PhyML, RAxML	Infer phylogenetic trees for nucleotide or amino acid sequences using either similarity or maximum likelihood-based algorithms
modelCompare, ProtTest	Determine which evolutionary model to use when constructing maximum likelihood trees
Archaeopteryx	Visualize, manipulate and decorate phylogenetic trees
Jmol	3D protein structure visualization / exploration
meta-CATS	Statistically compare groups of sequences to identify positions that significantly differ between them
BLAST	Identify similar nucleotide or amino acid sequences in a variety of custom ViPR databases
Sequence Variation Calculator	Compute the entropy present at each nucleotide or amino acid position at each position of user-defined groups of virus sequences
Short Peptide Identification Tool	Find short amino acid strings in target proteins using exact, fuzzy, or pattern matching
Genome Annotation Transfer Utility	Annotate a new genome sequence using an existing well-annotated reference genome
Primer3	Design PCR primers to amplify specific virus sequence(s) based on the data within ViPR

### 2.4. Workbench

The ViPR Workbench is a relatively unique feature that allows users to save search criteria and results, selected sequence records as working sets, and analysis results for future access. Any user can register for their own free private workspace for each virus family in the ViPR system simply by providing an email address and password to enable future log in. Working sets of nucleotide sequences can be converted into sets of amino acid sequences, and *vice versa*, using integrated sequence type conversion tools. The Workbench also facilitates the virtual sharing of user-selected content with collaborators around the world, regardless of their physical location. When saving search criteria in the Workbench, the system can automatically notify the user via email whenever new records that match the original query are added to the database. Selected sequence records from multiple searches can be merged using Boolean logic, and can be combined with user uploaded data to be analyzed with the integrated suite of tools in ViPR, while keeping the uploaded data private. Results from multiple sequence alignments, sequence variation analysis, and phylogenetic tree inferences can be saved for quick future retrieval.

### 2.5. Scientific Use Case

The ViPR team is constantly working to improve and extend the ViPR resource. Development of new system capabilities is frequently guided by scientific use cases that involve data access and storage approaches, novel analytical methods, and exploratory workflows that could be helpful to ViPR users. As an example of how the various types of data and metadata can be combined with the integrated analysis tools, we will employ one of these scientific use cases to demonstrate the utility of the ViPR system.

Recently, an analysis that characterized the sequence relationships between whole SARS-CoV genomes isolated from civets and humans was performed [8]. As an example use case, we have extended this previous study by performing an in-depth comparative genomics analysis of these sequences together with all other publicly available SARS-CoV sequences isolated from humans and civets between 2003 and 2004 using the capabilities and tools existing in the ViPR system. The bioinformatics workflow that was used follows these steps: (i) identify all whole genome sequence records of interest from human and civet through the *Coronaviridae*-specific search interface in ViPR, (ii) save the resulting sequence records as a Working Set in the integrated personal Workbench area of the resource, (iii) visualize the multiple sequence alignment, (iv) construct a maximum likelihood phylogenetic tree, (v) perform a metadata-driven comparative genomics statistical analysis of sequence variation between the host groups, and (vi) visualize where significantly differing residues are located on a 3D protein structure. 

#### 2.5.1. Searching for Relevant Sequence Records

We begin by searching for all SARS-CoV sequences in ViPR derived from isolates taken from either humans or civets. As of September 2012, this query returned 79 complete genome records consisting of 62 human records from 9 annotated countries and 17 civet records from 2 annotated countries isolated between 2003 and 2004, including strains taken during the height of the main and recurrent SARS-CoV outbreaks. Query results are displayed on the Genome Search Results page with contents sortable by clicking on the column headings. Desired records from the Search Results page can then be directly analyzed by selecting any of the integrated tools accessible as menu options under the “Run Analysis” pull-down tab, or saved as a working set in the users personal workbench. For the purposes of this use case, the sequence set was additionally filtered to remove fifteen genome records that did not have metadata for the year of isolation, two genome records that were derived from passaging of a virus strain already in the list, and one duplicate record for the RefSeq strain already in the list.

#### 2.5.2. Saving to the Workbench

All 61 genome sequence records matching the search and passing the filtering criteria were selected and saved to the Workbench for more in-depth analysis by clicking the “Add to Working Set” button on the Search Results page. This filtered dataset consisted of 15 isolates taken from civets and 46 isolates taken from humans.

#### 2.5.3. Performing a Multiple Sequence Alignment

**Figure 4:**
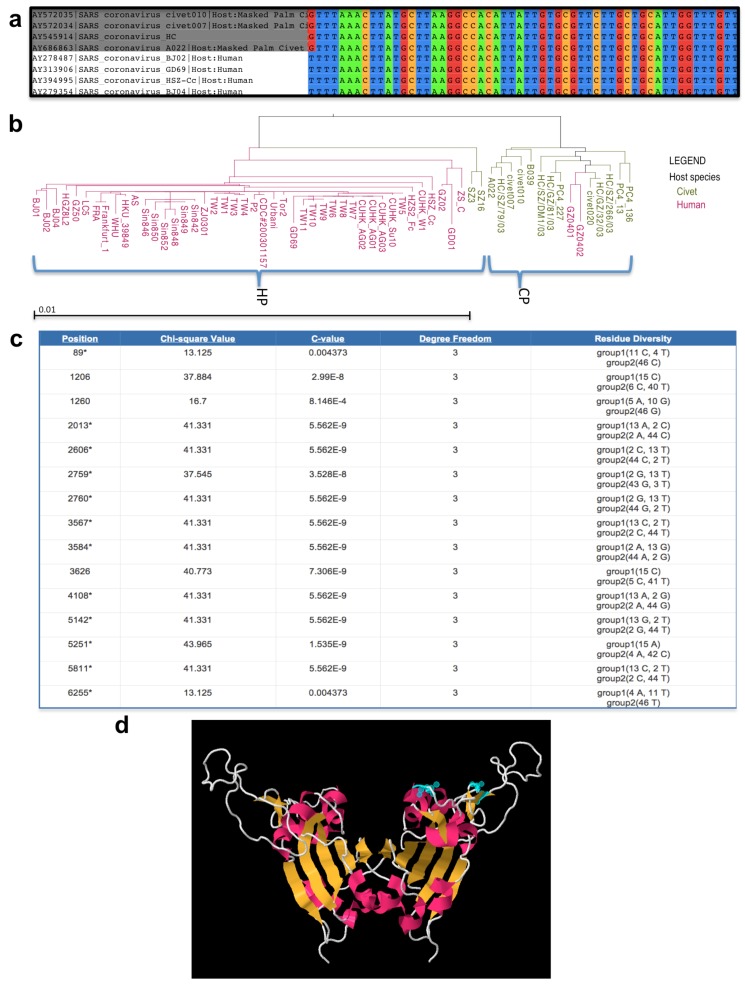
Scientific Use Case Comparing Human and Civet SARS-CoV Isolates. The results that were obtained from the various analytical and visualization tools provided in ViPR and explored in the scientific use case are shown. **(a)** A portion of a multiple sequence alignment of strains isolated from 2003 through 2004, from either humans (white labels) or civets (gray labels), including a column found to significantly differ between the specified groups based on meta-CATS analysis. **(b) **A Maximum Likelihood phylogenetic tree color-coded by host of isolation. Horizontal branch lengths are proportional to distance (the proportion of nucleotide changes). The distance scale for the proportion of changes is provided at the bottom of the panel. Strains belonging to the “civet-predominate” (CP) or “human-predominate” (HP) clades are delineated with blue braces. **(c)** An abridged table of the meta-CATS output containing significant positions, raw chi-square values, C-values (roughly equivalent to p-values), chi-square degrees of freedom and residue diversity for a subset of significant positions identified across the genome. **(d) **A 3D homodimeric protein structure of the SARS-CoV Spike protein showing secondary structure in cartoon and positions 479 and 487 (highlighted in blue on one of the monomeric chains), which were identified as significantly differing between hosts at the nucleotide level by meta-CATS.

Sequence data in FASTA format can be used as input for performing a fast customized multiple sequence alignment (MSA) in real-time. Data sources for this analysis can include search results, working sets and/or uploaded custom sequences. Sequences for RNA viruses are aligned with the MUSCLE algorithm on the ViPR server, with the ability to download the MSA results in FASTA format and/or save the MSA results to the Workbench. Once the alignment is finished, the integrated JalView tool allows the viewing and editing of the sequence and label information associated with the alignment to assist in interpreting the results. For the current use case, the nucleotide sequence alignment for the selected SARS-CoV strains confirms that these genomes are extremely well conserved across the entire length of the alignment even though they are derived from two very different host species (Figure 4a).

#### 2.5.4. Viewing and Exploring a Phylogenetic Tree

Nucleotide or amino acid sequence data can be used to construct phylogenetic trees in ViPR. Sequence data can be obtained from search results, working sets or custom data uploaded either to the Workbench or directly to the Phylogenetic Tree input page. Alternatively, phylogenetic trees can be constructed through the “Run Analysis” pull-down tab on the Multiple Sequence Alignment Results page directly. Once a phylogenetic tree is completed, the results can be saved in the Workbench or downloaded in the PhyloXML or Newick formats [44]. ViPR provides the FastME algorithm for constructing minimum evolution phylogenetic trees from the selected sequence data using the ‘Quick Tree’ option. The PhyML and RAxML maximum likelihood tree inferencing algorithms (with bootstrapping) have also been included in ViPR together with the modelCompare software to determine the evolutionary model best suited for use with any individual dataset.

When the phylogenetic tree construction is finished, the integrated Archaeopteryx phylogenetic tree viewer can be used to easily visualize, explore, interpret and manipulate the tree using built-in functions such as re-rooting, branch swapping, selecting sub-trees, etc. [38]. This visualization tool has been further customized to take advantage of the extensive metadata associated with the various sequence records stored in the ViPR database, to allow user-driven coloration of the labels at the terminal nodes (i.e. “leaves”) of the phylogenetic tree according to the host, country or year of isolation. A high-resolution image of the customized phylogenetic tree display can be exported in a variety of formats to enable more in-depth interpretation and/or inclusion in presentations or publications. For the current scientific use case, RAxML was used to reconstruct the phylogenetic tree for all sequence records that matched the original search and filtering criteria, which was subsequently colored based on the host of isolation (Figure 4b). The tree topology for this phylogenetic reconstruction shows two major clades that separate largely according to both the year and host of isolation. The majority of strains from the first clade were taken in 2003 with all but two members of this clade being isolated from civets (hereafter referred to as the ‘civet-predominate clade’). For the second clade, almost all of the strains were sampled in 2004 with all but two members isolated from humans (hereafter referred to as the ‘human-predominate clade’). This topology confirms that at least two species-jump events between civet and human have occurred [9]. It also suggests that this species barrier does not appear to be a significant bottleneck for SARS-CoV.

#### 2.5.5. Metadata-driven Comparative Analysis Tool for Sequences

The metadata-driven Comparative Analysis Tool for Sequences (meta-CATS) is an automated analysis workflow developed and implemented by the ViPR team to support the calculation of user-driven comparative genomics statistical analyses. This tool allows users to not only take advantage of the numerous sequence records in ViPR, but also the wealth of accompanying metadata for these records stored in the ViPR database. Such metadata may include date and/or geospatial point of specimen collection, host species, severity of disease, etc. Since this tool examines both the sequence and the associated metadata, statistically significant genotype-phenotype correlations can be detected. Input for this tool can include sequence search results, working sets, or custom upload of both sequence data and the associated metadata. Once the desired sequences are selected, the tool guides the user through the necessary steps of 1) assigning the sequences to up to five different groups based on metadata or other user criteria, 2) aligning all assigned sequences, 3) performing automated statistical analyses on the sequences, and 4) viewing the results.

For the current use case, the original sequence search results were automatically divided into two groups by the ViPR system based on the annotated host of isolation (human vs. civet). The meta-CATS results page displays the significant residues differing between the specified groups (Figure 4c), identifying 117 nucleotide positions in the entire SARS-CoV genome that significantly differed between the civet and human isolates. These positions were scattered throughout the genome and had calculated p-values ranging from 4.33x10^-12^ to 0.02492 (Supplementary Table 1). When this list was compared against the 26 nucleotide positions that differed between civet and human isolates reported in a previous study [8], all 26 of the previously identified positions were also found to be significant in this meta-CATS analysis.

#### 2.5.6. 3D Protein Structure Visualization and Exploration

ViPR has integrated the Jmol protein structure viewer application to facilitate the exploration and visualization of 3D protein structures either for custom uploaded data or for Protein Data Bank (PDB) structures from any taxa present in ViPR. The application has been enhanced to allow users to highlight active sites, immune epitopes and ligands, customize the appearance of the protein structure and quickly save an image or animated video of the structure. In addition, residues from the 3D protein structure(s) for each PDB file are mapped to the homologous positions in the stored UniProt records to facilitate quick and accurate comparison between structural data and amino acid sequence data.

For the current use case, we decided to explore the SARS-CoV Spike protein structure (PDB ID: 2GHV) in more detail since multiple residues were found to significantly differ between the civet and human isolates in the Spike coding region. Two of these significant nucleotide positions, 22942 and 22965, located in codons for amino acids 479 and 487, respectively, were particularly interesting since they were found to lie within the receptor-binding motif of the SARS-CoV receptor-binding domain of the Spike protein and had been reported to influence species-specific binding affinity for the host angiotensin-converting enzyme 2 (ACE2) [45]. The selected 3D structure includes most of the SARS-CoV receptor-binding domain of the Spike protein, which is located between residues 318-510 [46,47]. Secondary structures were displayed as ribbons and Spike amino acid positions 479 and 487 were highlighted in blue to confirm that they are exposed on the exterior surface of the protein in a position accessible for host receptor binding (Figure 4d). 

#### 2.5.7. Conclusions from Scientific Use Case

The workflow that was followed throughout the scientific use case confirms and extends results obtained through previous comparative genomics analyses and demonstrates the power of the ViPR system. Specifically, our phylogenetic tree corroborates the theory that there were at least two separate human outbreaks from viruses closely related to two separate clades isolated from civets during the time of the SARS-CoV epidemic. Our tree also shows a strong correlation between host and year of isolation, although this observation is not true for all strains included in the tree. The meta-CATS analysis identified significant p-values for 117 nucleotide residues that were identified as having significant variation between the civet and human isolates. The positions that are located within the SARS-CoV Spike protein receptor-binding motif, which were identified by meta-CATS, appear to reflect virus sequence variations that affect binding to host-specific receptor proteins. Additional wet-lab experimentation will be required to elucidate the specific function of the remaining significant sequence variations and whether they alter the fitness of the virus. Mapping residues and regions of interest onto a 3D protein structure can yield additional insight into their functional role. The scientific use case that was explored here serves as an example of the ability of ViPR to support data exploration and the generation of biologically relevant hypotheses that can then be subjected to more in-depth laboratory testing through experimentation.

## 3. Conclusions

The Virus Pathogen Database and Analysis Resource (ViPR, http://www.viprbrc.org), supported by the National Institute of Allergy and Infectious Diseases (NIAID) Bioinformatics Resource Centers (BRC) program, is a freely-available website that provides an intuitive search interface to access data about human pathogenic virus families, including *Coronaviridae,* obtained from public repositories, custom algorithms and direct-submission. These data are integrated with a suite of unique analytical and visualization tools for exploratory analysis. The ViPR resource provides researchers with an easy mechanism to not only perform complex analytical workflows, but to save the results and share them with collaborators to expedite discovery through the generation of experimentally-testable hypotheses. Such experimental discoveries can then be translated from the ‘bench’ to the ‘bedside’ in the form of diagnostics, prophylactics, vaccines and treatments for viruses belonging to the *Coronaviridae* family. 

## Supplementary Files

Supplementary File 1: PDF-Document (PDF, 15 KB)
