# Supplementary material for: Virus Pathogen Database and Analysis Resource (ViPR): A Comprehensive Bioinformatics Database and Analysis Resource for the Coronavirus Research Community
Source: Viruses. 2012 Nov 19;4(11):3209–26. doi: 10.3390/v4113209 (PMC3509690; doi:10.3390/v4113209)
Supplement: Supplementary File 1: — PDF-Document (PDF, 15 KB) [file viruses-04-03209-s001.pdf]

Aligned Positio	Sparse Chi-square Table (1=true, 0=false	Chi-square Valu	C-value	Degree Freedom	Residue Diversity (group1=civet, group2=humar		Reported by Shi, et al.	Spike Codon Number
89	1	13.125	0.004373	3	group1(11 C, 4 T)	group2(46 C)		
1206	0	37.884	2.99E-08	3	group1(15 C)	group2(6 C, 40 T)	Yes	
1260	0	16.7	8.15E-04	3	group1(5 A, 10 G)	group2(46 G)		
2013	1	41.331	5.56E-09	3	group1(13 A, 2 C)	group2(2 A, 44 C)		
2606	1	41.331	5.56E-09	3	group1(2 C, 13 T)	group2(44 C, 2 T)		
2759	1	37.545	3.53E-08	3	group1(2 G, 13 T)	group2(43 G, 3 T)		
2760	1	41.331	5.56E-09	3	group1(2 G, 13 T)	group2(44 G, 2 T)		
3567	1	41.331	5.56E-09	3	group1(13 C, 2 T)	group2(2 C, 44 T)		
3584	1	41.331	5.56E-09	3	group1(2 A, 13 G)	group2(44 A, 2 G)		
3626	0	40.773	7.31E-09	3	group1(15 C)	group2(5 C, 41 T)	Yes	
4108	1	41.331	5.56E-09	3	group1(13 A, 2 G)	group2(2 A, 44 G)		
5142	1	41.331	5.56E-09	3	group1(13 G, 2 T)	group2(2 G, 44 T)		
5251	1	43.965	1.54E-09	3	group1(15 A)	group2(4 A, 42 C)	Yes	
5811	1	41.331	5.56E-09	3	group1(13 C, 2 T)	group2(2 C, 44 T)		
6255	1	13.125	0.004373	3	group1(4 A, 11 T)	group2(46 T)		
6990	1	41.331	5.56E-09	3	group1(2 C, 13 T)	group2(44 C, 2 T)		
7137	1	41.331	5.56E-09	3	group1(2 C, 13 T)	group2(44 C, 2 T)		
7881	1	41.331	5.56E-09	3	group1(13 C, 2 T)	group2(2 C, 44 T)		
8502	1	43.965	1.54E-09	3	group1(15 G)	group2(4 G, 42 T)	Yes	
8559	0	40.773	7.31E-09	3	group1(15 C)	group2(5 C, 41 T)	Yes	
9176	1	43.965	1.54E-09	3	group1(15 C)	group2(4 C, 42 T)	Yes	
9335	1	41.331	5.56E-09	3	group1(13 C, 2 T)	group2(2 C, 44 T)		
9404	0	30.663	1.00E-06	3	group1(15 C)	group2(9 C, 37 T)	Yes	
9479	0	35.258	1.07E-07	3	group1(15 C)	group2(7 C, 39 T)	Yes	
9625	1	9.674	0.02155	3	group1(12 C, 3 T)	group2(46 C)		
10979	1	41.331	5.56E-09	3	group1(13 A, 2 T)	group2(2 A, 44 T)		
12121	1	41.331	5.56E-09	3	group1(13 C, 2 T)	group2(2 C, 44 T)		
13762	1	41.331	5.56E-09	3	group1(13 G, 2 T)	group2(2 G, 44 T)		
14120	1	41.431	2.19E-08	4	group1(12 A, 2 G, 1 N)	group2(2 A, 44 G)		
14304	1	41.331	5.56E-09	3	group1(13 C, 2 T)	group2(2 C, 44 T)		
17378	1	19.802	1.87E-04	3	group1(7 C, 8 T)	group2(44 C, 2 T)		
17394	0	24.246	2.22E-05	3	group1(8 C, 7 T)	group2(46 C)		
17568	0	23.441	3.27E-05	3	group1(15 G)	group2(13 G, 33 T)	Yes	
18184	1	45.66	6.70E-10	3	group1(2 A, 13 C)	group2(45 A, 1 C)		
19410	1	41.331	5.56E-09	3	group1(2 A, 13 G)	group2(44 A, 2 G)		
20854	0	40.773	7.31E-09	3	group1(15 A)	group2(5 A, 41 G)	Yes	
21246	1	13.125	0.004373	3	group1(11 C, 4 T)	group2(46 C)		
21735	0	26.774	6.57E-06	3	group1(15 A)	group2(11 A, 35 G)	Yes	77
21921	1	41.331	5.56E-09	3	group1(2 C, 13 T)	group2(44 C, 2 T)		139
21945	1	19.802	1.87E-04	3	group1(7 A, 8 G)	group2(44 A, 2 G)		147
22186	1	16.697	8.16E-04	3	group1(2 A, 10 C, 3 G)	group2(46 C)		227
22233	1	13.125	0.004373	3	group1(11 A, 4 G)	group2(46 A)		243
22236	0	23.441	3.27E-05	3	group1(15 C)	group2(13 C, 33 T)	Yes	244
22531	0	37.884	2.99E-08	3	group1(15 G)	group2(40 A, 6 G)	Yes	342
22536	0	40.773	7.31E-09	3	group1(15 G)	group2(41 A, 5 G)	Yes	344
22584	1	51.477	3.87E-11	3	group1(15 C)	group2(2 C, 44 T)	Yes	360

22888	1	41.331	5.56E-09	3	group1(2 C, 13 T)	group2(44 C, 2 T)		461
22889	0	16.7	8.15E-04	3	group1(10 C, 5 T)	group2(46 C)		462
22920	1	41.331	5.56E-09	3	group1(13 C, 2 T)	group2(2 C, 44 T)		472
22941	0	32.372	4.37E-07	3	group1(6 A, 9 G)	group2(46 A)		479
22942	1	41.149	6.08E-09	3	group1(11 A, 4 T)	group2(46 T)		479
22944	1	41.331	5.56E-09	3	group1(2 A, 13 G)	group2(44 A, 2 G)		480
22965	1	51.477	3.87E-11	3	group1(15 G)	group2(44 C, 2 G)	Yes	487
23330	1	45.66	6.70E-10	3	group1(2 G, 13 T)	group2(45 G, 1 T)		609
23331	1	45.66	6.70E-10	3	group1(2 C, 13 T)	group2(45 C, 1 T)		609
23344	1	45.66	6.70E-10	3	group1(13 A, 2 T)	group2(1 A, 45 T)		613
23499	1	51.477	3.87E-11	3	group1(15 C)	group2(2 C, 44 T)	Yes	665
23733	1	41.331	5.56E-09	3	group1(2 C, 13 G)	group2(44 C, 2 G)		743
23799	1	41.331	5.56E-09	3	group1(2 C, 13 T)	group2(44 C, 2 T)		765
23837	0	35.258	1.07E-07	3	group1(15 G)	group2(7 G, 39 T)	Yes	778
24397	1	9.674	0.02155	3	group1(12 G, 3 T)	group2(46 G)		964
24580	0	40.773	7.31E-09	3	group1(15 C)	group2(5 C, 41 T)	Yes	1025
24744	1	9.674	0.02155	3	group1(12 A, 3 G)	group2(46 A)		1080
24992	1	43.965	1.54E-09	3	group1(15 G)	group2(42 A, 4 G)	Yes	1163
25045	1	41.331	5.56E-09	3	group1(13 C, 2 T)	group2(2 C, 44 T)		1180
25300	1	51.477	3.87E-11	3	group1(15 A)	group2(2 A, 44 T)	Yes	
25355	1	36.741	2.04E-07	4	group1(11 A, 3 C, 1 T)	group2(2 A, 43 C, 1 Y)		
25370	1	41.331	5.56E-09	3	group1(2 A, 13 G)	group2(44 A, 2 G)		
25522	1	55.937	4.33E-12	3	group1(15 A)	group2(1 A, 45 T)	Yes	
25642	1	51.477	3.87E-11	3	group1(15 G)	group2(2 G, 44 T)	Yes	
25707	1	45.66	6.70E-10	3	group1(13 A, 2 T)	group2(1 A, 45 T)		
26057	1	9.674	0.02155	3	group1(3 C, 12 T)	group2(46 T)		
26424	1	51.477	3.87E-11	3	group1(15 A)	group2(2 A, 44 G)	Yes	
26450	1	41.331	5.56E-09	3	group1(2 A, 13 G)	group2(44 A, 2 G)		
26600	1	43.965	1.54E-09	3	group1(15 C)	group2(4 C, 42 T)	Yes	
27199	1	9.674	0.02155	3	group1(12 A, 3 G)	group2(46 A)		
27400	1	23.536	3.12E-05	3	group1(7 A, 8 G)	group2(45 A, 1 G)		
27439	1	36.606	5.58E-08	3	group1(3 C, 12 T)	group2(44 C, 2 T)		
27841	1	23.439	1.04E-04	4	group1(15 C)	group2(2 -, 13 C, 31 T)	Yes	
27897	0	40.773	3.00E-08	4	group1(15 T)	group2(41 -, 5 T)		
27898	0	40.773	3.00E-08	4	group1(15 C)	group2(41 -, 5 C)		
27899	0	40.773	3.00E-08	4	group1(15 C)	group2(41 -, 5 C)		
27900	0	40.773	3.00E-08	4	group1(15 T)	group2(41 -, 5 T)		
27901	0	40.773	3.00E-08	4	group1(15 A)	group2(41 -, 5 A)		
27902	0	40.773	3.00E-08	4	group1(15 C)	group2(41 -, 5 C)		
27903	0	40.773	3.00E-08	4	group1(15 T)	group2(41 -, 5 T)		
27904	0	40.773	3.00E-08	4	group1(15 G)	group2(41 -, 5 G)		
27905	0	40.773	3.00E-08	4	group1(15 G)	group2(41 -, 5 G)		
27906	0	40.773	3.00E-08	4	group1(15 T)	group2(41 -, 5 T)		
27907	0	40.773	3.00E-08	4	group1(15 T)	group2(41 -, 5 T)		
27908	0	40.773	3.00E-08	4	group1(15 A)	group2(41 -, 5 A)		
27909	0	40.773	3.00E-08	4	group1(15 C)	group2(41 -, 5 C)		
27910	0	40.773	3.00E-08	4	group1(15 C)	group2(41 -, 5 C)		

27911	0	40.773	3.00E-08	4	group1(15 A)	group2(41 -, 5 A)
27912	0	40.773	3.00E-08	4	group1(15 A)	group2(41 -, 5 A)
27913	0	40.773	3.00E-08	4	group1(15 C)	group2(41 -, 5 C)
27914	0	40.773	3.00E-08	4	group1(15 C)	group2(41 -, 5 C)
27915	0	40.773	3.00E-08	4	group1(15 T)	group2(41 -, 5 T)
27916	0	40.773	3.00E-08	4	group1(15 G)	group2(41 -, 5 G)
27917	0	40.773	3.00E-08	4	group1(15 A)	group2(41 -, 5 A)
27918	0	40.773	3.00E-08	4	group1(15 A)	group2(41 -, 5 A)
27919	0	40.773	3.00E-08	4	group1(15 T)	group2(41 -, 5 T)
27920	0	40.773	3.00E-08	4	group1(15 G)	group2(41 -, 5 G)
27921	0	40.773	3.00E-08	4	group1(15 G)	group2(41 -, 5 G)
27922	0	40.773	3.00E-08	4	group1(15 A)	group2(41 -, 5 A)
27923	1	31.239	2.74E-06	4	group1(2 -, 13 A)	group2(41 -, 5 A)
27924	1	31.239	2.74E-06	4	group1(2 -, 13 T)	group2(41 -, 5 T)
27925	0	40.773	3.00E-08	4	group1(15 A)	group2(41 -, 5 A)
27936	1	41.35	2.28E-08	4	group1(13 A, 2 C)	group2(2 -, 2 A, 42 C)
28145	1	12.774	0.01244	4	group1(9 A, 6 C)	group2(1 -, 43 A, 2 C)
28243	1	45.66	6.70E-10	3	group1(13 C, 2 T)	group2(1 C, 45 T)
28633	1	9.671	0.02158	3	group1(12 C, 1 G, 2 T)	group2(46 C)
28993	1	9.355	0.02492	3	group1(10 A, 5 G)	group2(44 A, 2 G)
29052	1	9.355	0.02492	3	group1(10 C, 5 T)	group2(44 C, 2 T)
29065	1	41.331	5.56E-09	3	group1(13 G, 2 T)	group2(2 G, 44 T)
29313	1	9.674	0.02155	3	group1(12 C, 3 T)	group2(46 C)
29768	1	23.524	3.14E-05	3	group1(5 C, 3 G)	group2(1 C, 42 G)
